# Evaluation of MWCNTs as Nanoadditives in a Propanol–Biodiesel–Diesel Mixture on Compression Ignition Engine Characteristics

**DOI:** 10.1021/acsomega.5c10610

**Published:** 2026-01-22

**Authors:** Yahya Çelebi, Mazlum Cengiz, Ahmet Aydın, Arantzazu Gómez, Reyes García-Contreras

**Affiliations:** † 187477 Şırnak University, Department of Motor Vehicles and Transportation Technologies, Sirnak 73000, Turkiye; ‡ Department of Machinery and Metal Technologies, Şırnak University, Sirnak 73000, Turkiye; § Department of Mechatronics Engineering, Nişantaşı University, Istanbul 34453, Turkiye; ∥ School of Industrial and Aerospace Engineering, Castilla-La Mancha University, Toledo 45071, Spain

## Abstract

Cleaner alternatives to internal combustion engines are essential for reducing pollution emissions while maintaining performance. In this study, a ternary blend of diesel, biodiesel, and propanol (80:10:10 by volume) was prepared and enriched with multiwalled carbon nanotubes (MWCNTs) at concentrations of 40, 60, and 80 ppm to evaluate its combustion, performance, and emission in a monocylinder diesel engine. Compared to diesel fuel, the ternary blends with MWCNTs demonstrated an increase of up to 6.73% in the net heat release rate and an average decrease of up to 14.74% in cylinder pressure. Engine performance improved with a rise in the brake thermal efficiency up to 11.79% on average, while the brake-specific fuel consumption decreased by up to an average of 6.04%. Emission analysis indicated substantial reductions in carbon dioxide (up to 18.72%) and nitrogen oxides (up to 42.39%), whereas unburned hydrocarbons increased by an average of 26.66%. These results indicate that the synergistic interaction among biodiesel oxygen content, propanol volatility, and the catalytic/thermal conductivity properties of MWCNTs enables improved combustion efficiency while mitigating carbon dioxide and nitrogen oxides. This study provides new insight into the role of carbon-based nanomaterials in enhancing oxygenated ternary diesel blends and highlights their potential to support cleaner and more efficient compression ignition engine operation.

## Graphical abstract

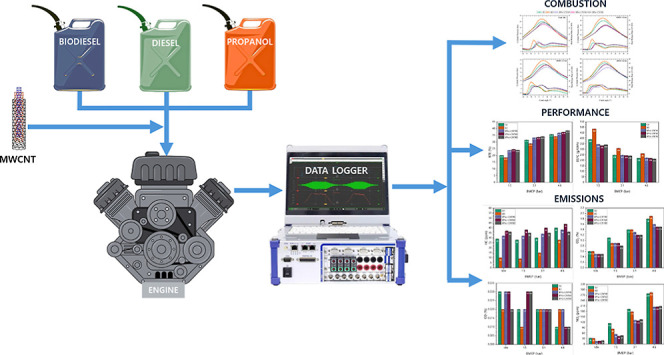

## 1 Introduction

Global energy consumption has maintained an upward trajectory (except during the COVID-19 pandemic in the late 2019 to 2020 period), driven by expanding populations, technological innovations, industrial expansion, urban development, and enhanced living conditions.^1^ This trend increases energy demand and contributes to environmental challenges such as ozone layer depletion, acid rain, and atmospheric pollution,^2^ posing a serious threat to both living organisms and the broader ecosystem.^3^ Furthermore, it forces governments, industries, policymakers, and research institutions to develop long-term strategies to achieve carbon neutrality and pursue innovative and sustainable solutions, including renewable energy technologies and alternative fuel systems, to ensure efficient and clean energy production.^4^

The transportation industry represents a substantial share of worldwide energy usage, predominantly sourced from petroleum-based fuels. Besides, it is responsible for over 25% of global greenhouse gas emissions,^5^ and compression ignition engines (CIEs) alone contribute to 70% of overall vehicle emissions.^6^ Over 92% of worldwide fossil fuel consumption, comprising diesel and gasoline, is attributed to road transportation networks.^7^ Even though hybrid and electrical vehicles (EVs) have been becoming more widespread worldwide,^8^ CIEs remain prevalent in transportation services and industrial sectors^9^ because of their remarkable durability, longevity, lower maintenance,^10^ superior thermal efficiency,^11^ higher fuel economy,^12^ and substantial torque production.^13^ Also, EVs are not completely carbon-neutral because their batteries are neither manufactured from sustainable resources^14^ nor fully recycled. Therefore, until EVs are entirely developed using renewable resources, alternative fuels for CIEs should be explored, as crude oil is a nonrenewable resource that is gradually depleting and has already prompted extensive research on fuel alternatives.^15^

In this way, hydrogen, biogas, compressed natural gas, and biodiesel produced from vegetable oils or recycled waste oils have become a promising option due to their nontoxic and eco-friendly properties,^16^ and it reduces reliance on global crude oil.^17^ Additionally, they have a lower carbon footprint compared to conventional diesel fuel. Pure biodiesel (BD) and its mixtures with diesel fuel are compatible with existing CIEs, typically requiring no structural changes.^18,19^ The oxygen present in biodiesel lowers exhaust emissions, such as hydrocarbons (HC), carbon monoxide (CO), soot, and particulate matter (PM), as well as improves combustion efficiency.^20,21^ On the contrary, biodiesel is constrained by certain drawbacks such as higher viscosity^8^ and reduced calorific value.^22^ Furthermore, the application of biodiesel as a direct replacement fuel in conventional diesel results in elevated nitrogen oxides (NO_*x*_) emissions.^23^ Therefore, the application of BD or its mixtures with conventional diesel is insufficient to achieve the desired engine performance and emission levels. Higher alcohols, including propanol, hexanol, butanol, and pentanol, can be blended with biodiesel to decrease its viscosity and density, leading to shorter spray tip penetration and narrower cone angles, thereby bringing its spray characteristics closer to those of conventional diesel.^24^

Alternatively, Reactivity-Controlled Compression Ignition (RCCI) and Homogeneous Charge Compression Ignition (HCCI) engines, which possess high thermal efficiency, were designed to reduce NO_*x*_ and smoke opacity; however, they have a limited operation range for different engine loads and speeds. Also, their combustion modes rely on the fuels’ chemical kinetics, and their combustion process cannot be controlled by a physical mechanism.^25^

As another alternative, nanoparticles have been added to liquid fuels to enhance fuel characteristics, thus enhancing the performance, combustion quality, and emission characteristics of ICEs,^26^ as well as prolonging their lifespan,^15,27^ provided that the blend ratios are carefully adjusted with appropriate nanoparticles while addressing compatibility and stability concerns.^10^ In addition, the inclusion of nanoparticles improves oxidation and fuel evaporation due to their large surface-to-volume ratio and decreases the ignition delay time on account of their thermal conductivity.^28^ Nanoparticles are generally divided into four categories: organic, inorganic, carbon, and composite-based.^27^ Particularly, carbon-based nanoparticles offer more advantages compared to regular metal additives in terms of thermal conductivity,^29^ mechanical strength, and eco-compatibility.^8^ Additionally, the development and potential release of metal oxides and metal nanoparticles into the environment may cause ecological issues.^30^ For example, Sathish et al.^31^ added multi-walled carbon nanotubes (MWCNTs) at three different fractions of 40 ppm/L, 80 ppm/L, and 120 ppm/L into a 20% biodiesel-80% diesel blend to enhance its combustion and exhaust emission characteristics as an alternative fuel for diesel engines. The inclusion of MWCNTs at all selected fractions in the blend improved the brake thermal efficiency (BTE), lowered brake-specific fuel consumption (BSFC), and reduced NO*x* and CO emissions. Especially, addition of 120 ppm fraction delivered the most favorable outcomes, attaining a peak BTE of 30.19%, the lowest BSFC of 0.282 kg/kW-h, and remarkably reduced harmful pollutants, with NO*x*, HC, CO, and smoke emissions measured at 0.052%, 52%, 11 ppm, and 982 ppm, respectively. Likewise, Ooi et al.^32^ evaluated the influence of 25, 50, and 100 ppm of MWCNTs’ inclusion into a 20% palm oil-based biodiesel and 80% diesel blend in terms of efficiency, combustion, and harmful emissions in a monocylinder direct injection CIE at various engine speeds of 1500, 2000, and 2500 rpm. The addition of MWCNTs decreased the ignition delay by up to 17.6%, improved combustion phasing as high as 12.9%, and shortened the combustion duration by 18.5%. Furthermore, it enhanced the BSFC by 15.7% and the BTE by as high as 16.3%. Moreover, it improved CO and HC emissions, reaching 34.7% and 16%, respectively. On the other hand, it worsened NO_*x*_ emissions, reaching as high as 43.5%. Consequently, the MWCNTs’ inclusion into biodiesel–diesel blends could improve the efficiency, combustion, and exhaust emission traits of the diesel engine.

Although several studies have reported the use of propanol–diesel blends and others have examined MWCNT additives in biodiesel–diesel mixtures, the combined use of diesel, biodiesel, and propanol together with MWCNTs in a ternary fuel has not been systematically addressed in the literature. The present study specifically investigates a diesel–biodiesel–propanol blend (80:10:10 by volume) enhanced with MWCNTs and evaluates its combustion, performance, and emission behavior under various engine loads. MWCNTs were added to the blends at concentrations of 40, 60, and 80 ppm per liter. This work therefore contributes new insights into the combined synergistic effects of oxygenated alcohol, biodiesel, and carbon nanomaterials within the same fuel system. The aim of this fuel formulation is to reduce diesel fuel proportions by using promising biofuels, such as propanol and biodiesel, while improving engine exhaust emissions.

## 2 Materials and Methodology

### 2.1 Fuel Preparation and Characterization

Ultra low sulfur diesel (D2) was used for this experimental research to prepare the ternary blend and also served as baseline fuel for base comparisons. BD was produced from soya oil. Both biodiesel and propanol were obtained from a commercial company. To enhance the fuel properties of a ternary blend consisting of diesel, propanol, and biodiesel (80:10:10 by volume), MWCNTs were introduced in concentrations of 40, 60, and 80 ppm per liter and named as BPro + CNT40, BPro + CNT60, and BPro + CNT80, respectively. To ensure homogeneity of the test fuels, an ultrasonic mixer operating at 24 kHz and a constant temperature of 25 ± 2 °C for 30 min was used. The fuel properties of test samples used in this experimental research are shown in Table 1.

**Table 1:** Main Properties of the Tested Fuels

Characteristic	D2	2-Propanol	BD	BPro + CNT40	BPro + CNT60	BPro + CNT80
Heating value (kJ/kg)	45978	24040^a^	40384	43850	43887	43912
Density (kg/m^3^) at 15 °C	798	781^a^	850	785	786	786
Pour point (°C)	>-20	-	–1.7	>-20	>-20	>-20
Freezing point (°C)	>-20	-	–2	>-20	>-20	>-20
Cloud point (°C)	–7	-	1	-	-	-
Viscosity (mm^2^/s) at 40 °C	2.91	1.33^a^	4.73	2.36	2.39	2.46
Cetane number	52	12^d^	54.6	48.2	48.2	48.2
Latent heat of evaporation (kJ/kg)	251^b^	756^c^	238^b^	-	-	-

a Data taken from ref 33.

b Data taken from ref 34.

c Data taken from ref 35.

d Data taken from ref 36.

The 80:10:10 blending proportion was selected based on miscibility, stability observations, and combustion behavior reported in prior studies. Higher alcohol contents (>10 vol %) tend to cause cooling effects and prolonged ignition delay due to the high latent heat of vaporization of propanol, which leads to incomplete combustion and starting difficulties.^37^ Similarly, biodiesel contents beyond 10 vol % significantly increase viscosity and density, impairing spray formation and raising in-cylinder temperatures, which may elevate NO_*x*_.^38^ In preliminary tests, the 80:10:10 mixture exhibited full miscibility without surfactants, stable dispersion of MWCNT nanoparticles, and combustion characteristics closest to those of diesel operation.

#### 2.1.1 Nanomaterial Analysis

Figures 1 and 2 display a scanning electron microscopy (SEM) EDX image and different transmission electron microscopy (TEM) images of the tested MWCNT particles, respectively.^39^

**Figure 1:**
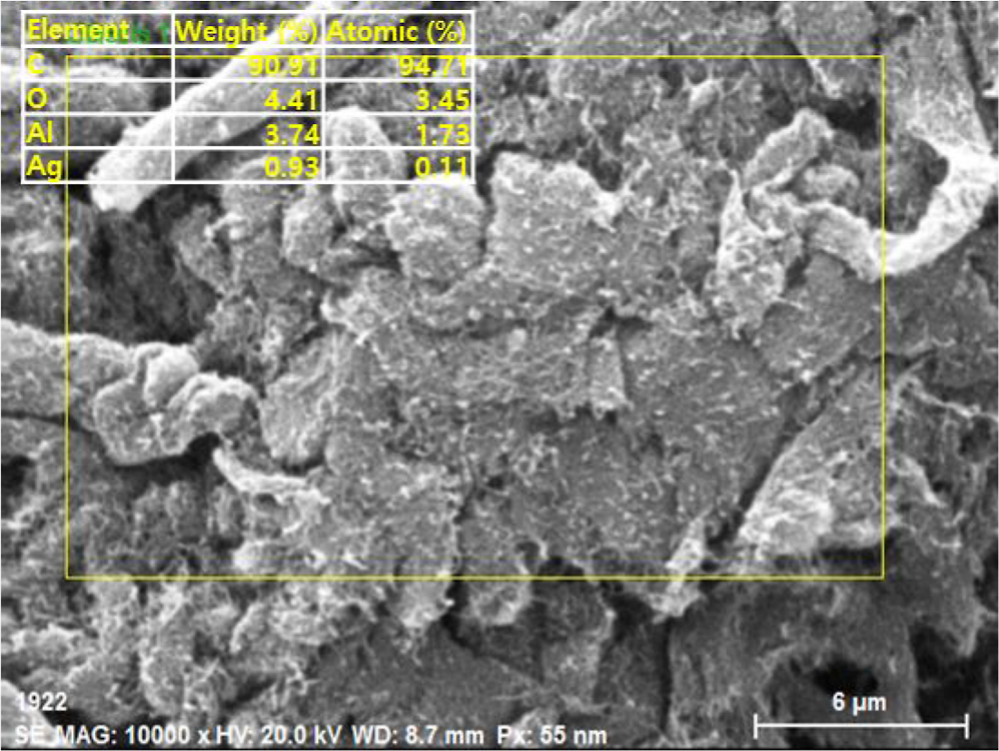
SEM EDX visuals for the tested MWCNT particles.

**Figure 2:**
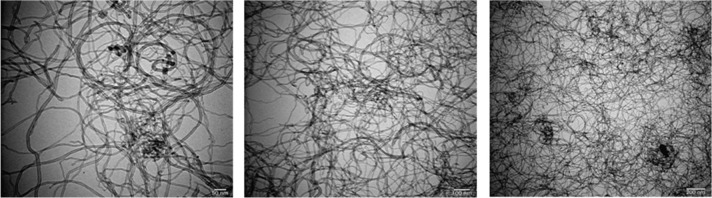
TEM visuals of MWCNT powder.

MWCNTs are produced using the chemical vapor deposition (CVD) method and exhibit a black color. They have an outside diameter ranging from 8 to 10 nm, an inside diameter of 5 to 15 nm, and a length of 1 to 3 μm. The material has a compressed density of 0.25 g/cm^3^ and an actual density of 2.4 g/cm^3^. Its ash content is 1.5% weight, with no specified COOH content. The MWCNTs demonstrate an electrical conductivity of 98 S/cm, a thermal conductivity of 3000 W/mK,^40^ and a specific surface area of 290 m^2^/g.

Figure 3 displays the thermogravimetric (TG) and differential thermal analysis (DTA) profiles for MWCNTs as a function of temperature, ranging from 0 to 800 °C.^39^ The TG analysis was conducted to measure weight change as a function of temperature. The TG curve shows a gradual mass loss starting from around 100 °C, with a significant decline beginning around 400 °C and dropping to approximately 91% by 800 °C, indicating thermal decomposition. The DTA was carried out to investigate the thermal behavior of the material. The DTA curve exhibits an endothermic peak around 450–500 °C, suggesting a phase transition or energy absorption during this temperature range. The mass percentage remains relatively stable above 98% until around 400 °C, after which it decreases steadily. TGA and DTA demonstrate the thermal stability of MWCNTs at high temperatures, confirming that the nanotubes remain structurally stable throughout the combustion process and can, therefore, participate in catalytic heat transfer enhancement.

**Figure 3:**
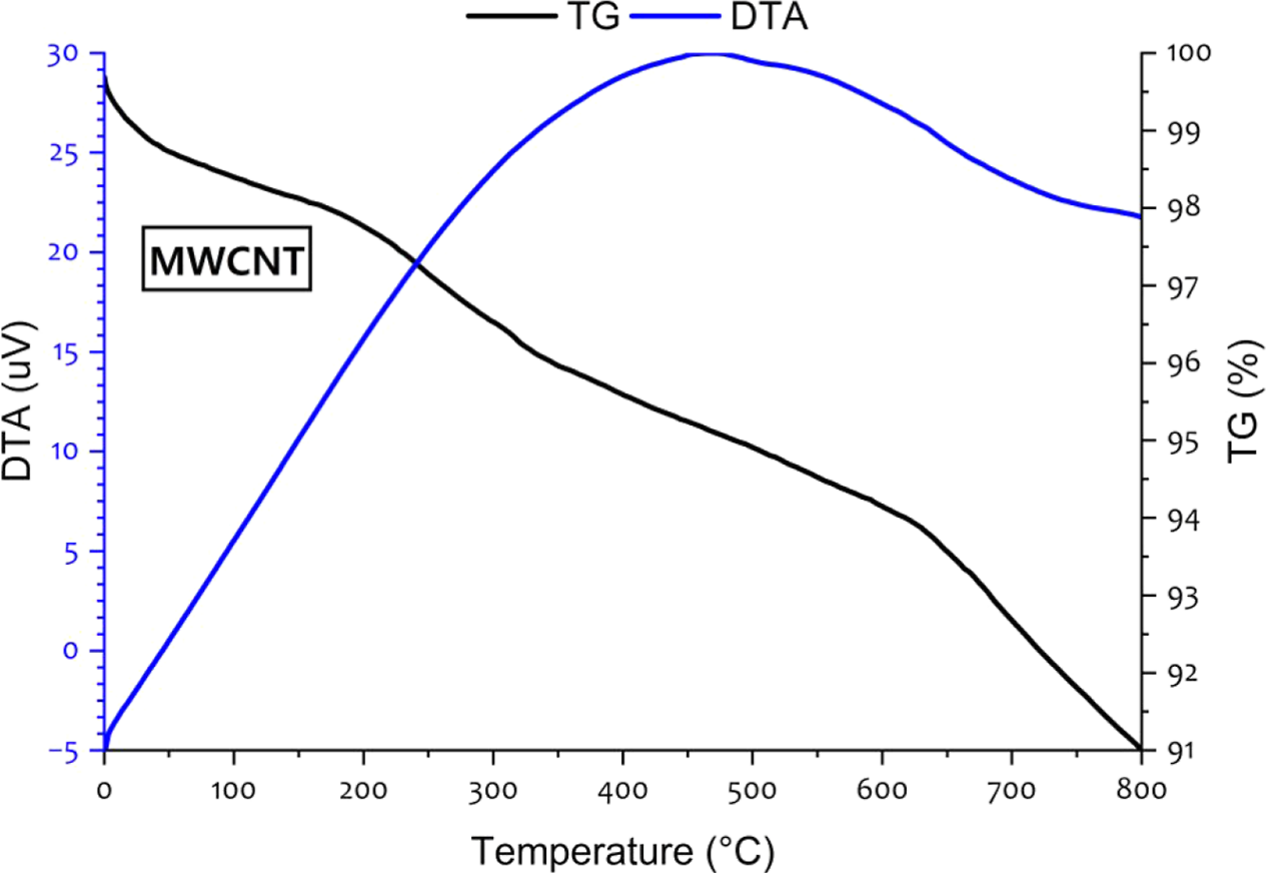
TGA behavior for MWCNT nanoparticles.

### 2.2 Physicochemical Characterization

#### 2.2.1 GC–MS Analysis

Table 2 presents the GC–MS analysis results, showing the relative percentages of fatty acids in soya oil and biodiesel.^39^ The most striking compositional shift occurs in the major unsaturated fatty acids: oleic acid (C18:1) increases dramatically from 26% in soya oil to 51% in biodiesel, while linoleic acid (C18:2) decreases substantially from 52% in soya oil to 23% in biodiesel, representing an apparent conversion or rearrangement during the biodiesel production process. The saturated fatty acid profile shows more modest changes, with palmitic acid (C16:0) increasing slightly from 11% to 14% and stearic acid (C18:0) remaining relatively stable at 4.5% to 5.3%. Minor fatty acid components, including the long-chain saturated acids (arachidic and behenic) and the less common unsaturated acids (palmitoleic, heptadecanoic, *cis*-11-eicosenoic, and gamma linolenic), remain at trace levels below 1% in both samples. This compositional transformation suggests that the transesterification process may involve selective reactions or isomerization that favor the formation of monounsaturated over polyunsaturated fatty acid methyl esters, potentially improving the oxidative stability of the resulting biodiesel by reducing the proportion of highly unsaturated linoleic acid while increasing the more stable oleic acid content.

**Table 2:** GC–MS Analysis of Soya Oil and Its Derived Biodiesel

Fatty acids	Biodiesel (% wt)	Soya oil (% wt)
Arachidic acid	0.51	0.38
Behenic acid	0.32	0.43
Cis-11-eicosenoic acid	0.83	0.18
Gamma linolenic acid	3.71	6.01
Heptadecanoic acid	0.27	0.08
Linoleic acid	23.15	51.53
Myristic acid	0.46	0
Oleic acid	50.85	25.85
Palmitic acid	13.95	10.93
Palmitoleic acid	0.60	0.10
Stearic acid	5.34	4.50

#### 2.2.2 FTIR Analysis

Figure 4 displays the Fourier transform infrared spectroscopy (FTIR) analysis results for the produced soya vegetable oil biodiesel.^39^ This FTIR spectrum demonstrates the comparative molecular vibrational analysis of raw oil and biodiesel through transmittance measurements across the mid-infrared region (500–4000 cm^–1^). The spectral data reveal characteristic absorption bands corresponding to specific molecular vibrations: the intense peak at 1742.7 cm^–1^ represents the C=O stretching vibration of ester carbonyl groups, confirming the presence of triglyceride esters in raw oil and methyl/ethyl esters in biodiesel; the dual peaks at 2853.1 and 2922.2 cm^–1^ correspond to symmetric and asymmetric C–H stretching vibrations of aliphatic methyl and methylene groups in fatty acid chains; the absorption at 1435.8 cm^–1^ indicates C–H bending (scissoring) vibrations of CH_2_ groups; the peak at 1158.9 cm^–1^ represents C–O stretching vibrations of the ester linkage; and the band at 721.75 cm^–1^ corresponds to CH_2_ rocking vibrations in long-chain alkyl groups. The peak at 3004.1 cm^–1^ likely represents the =C–H stretching of unsaturated bonds in the fatty acid chains. The nearly superimposable spectra indicate that transesterification has occurred without significant alteration to the fundamental molecular structure of the fatty acid chains, with the primary difference being the conversion from glycerol-based triglycerides to lower-molecular-weight alkyl esters. The absence of a broad O–H stretching around 3200–3600 cm^–1^ confirms the absence of significant water or alcohol contamination, and the maintained ester functionality validates successful biodiesel synthesis while preserving the fatty acid profile.

**Figure 4:**
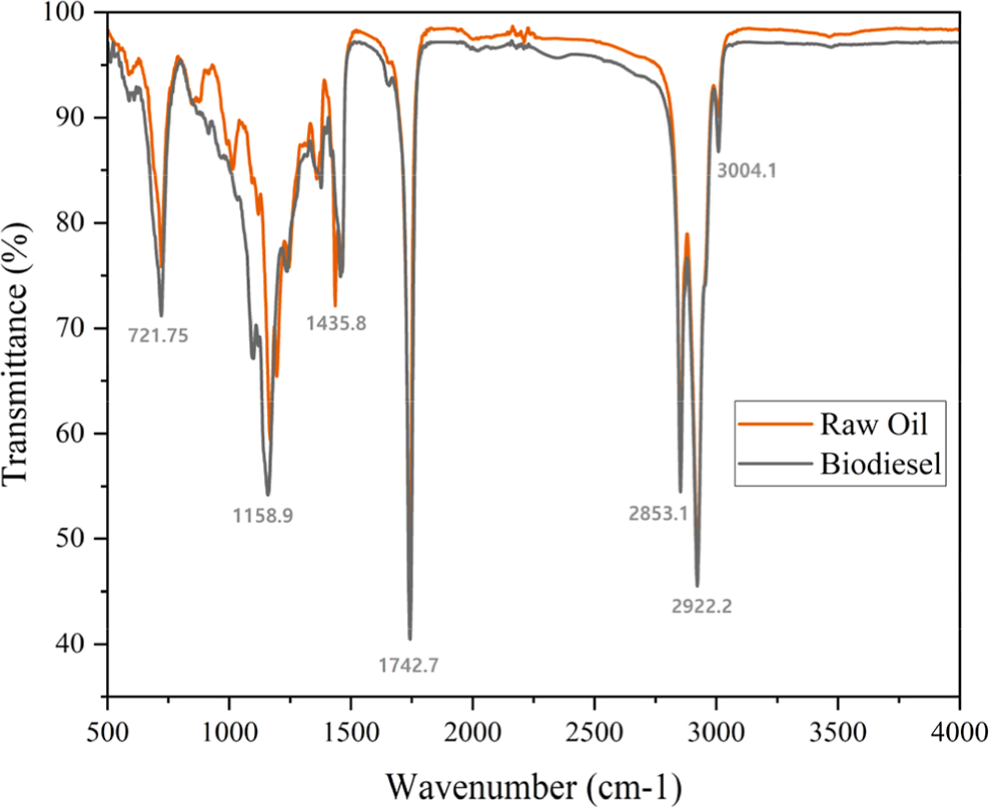
Comparative FT-IR spectra of soya oil and its derived biodiesel.

#### 2.2.3 NMR Analysis

Figure 5 shows the comparative ^1^H-NMR (proton nuclear magnetic resonance) spectrum of soya oil and its derived biodiesel.^39^ This comparative ^1^H-NMR analysis reveals the structural differences between biodiesel and soya oil at the molecular level through characteristic chemical shift patterns. In the biodiesel spectrum, the distinctive singlet at approximately 3.7 ppm represents the methoxy protons (−OCH_3_) of fatty acid methyl esters (FAME), which is the diagnostic peak confirming successful transesterification from triglycerides to methyl esters. Both spectra show similar aliphatic proton patterns in the 0.8–2.6 ppm region, including the terminal methyl groups of fatty acid chains (0.8–0.9 ppm), bulk methylene protons (1.2–1.3 ppm), β-methylene protons adjacent to carbonyl groups (1.6 ppm), and α-methylene protons (2.3 ppm). The soya oil spectrum displays characteristic glycerol backbone protons around 4.1–4.3 ppm (glycerol –CH_2_– protons) and 5.2 ppm (central glycerol −CH-proton), which are absent in the biodiesel spectrum, confirming the removal of glycerol during transesterification. Both spectra exhibit peaks around 5.3 ppm corresponding to olefinic protons (=CH) from unsaturated fatty acids, indicating preservation of the double bond structure during the conversion process. The integration patterns and chemical shift assignments provide definitive structural confirmation of the conversion from triglyceride (soya oil) to fatty acid methyl esters (biodiesel), with the methoxy peak serving as the key identifier for successful biodiesel production.

**Figure 5:**
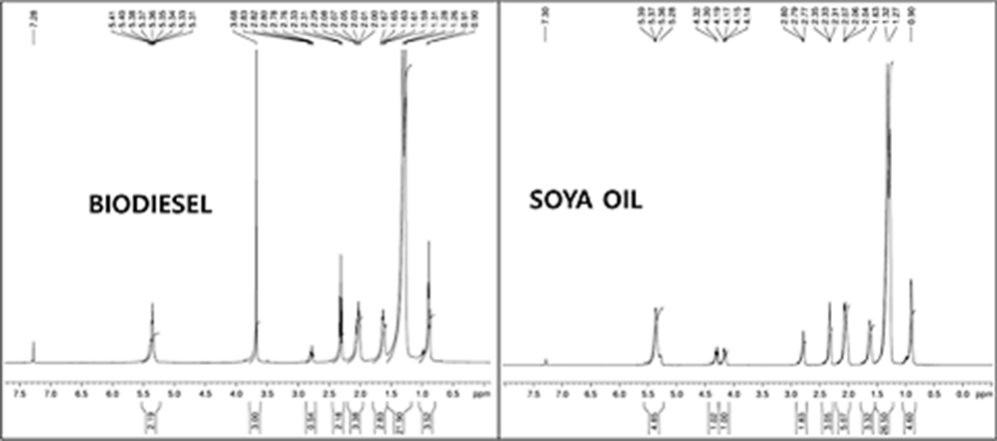
Comparative ^1^H-NMR spectrum of soya oil and its derived biodiesel.

Figure 6 illustrates the comparative ^13^C-NMR (carbon-13 nuclear magnetic resonance) spectrum of soya oil and its derived biodiesel.^39^ The most diagnostic difference appears in the carbonyl region around 170–180 ppm, where biodiesel shows a single carbonyl carbon peak at approximately 174 ppm corresponding to the ester carbonyl of fatty acid methyl esters, while soya oil exhibits carbonyl carbons around 172–173 ppm from the triglyceride ester linkages. The critical structural distinction is evident in the 50–70 ppm region: biodiesel displays a characteristic peak at approximately 51 ppm representing the methoxy carbon (−OCH_3_) of methyl esters, which is completely absent in the soya oil spectrum, confirming successful transesterification. Conversely, soya oil shows distinctive glycerol backbone carbons at around 62 ppm (terminal –CH_2_OH carbons) and 69 ppm (central –CHOH carbon) that are eliminated in the biodiesel spectrum. Both spectra share similar aliphatic carbon patterns in the 10–40 ppm region, including terminal methyl carbons (14 ppm), bulk methylene carbons (22–34 ppm), and carbons α and β to the carbonyl group, indicating preservation of the fatty acid chain structure. The olefinic carbons around 127–130 ppm are present in both spectra, confirming retention of unsaturation during the conversion process. This ^13^C NMR data provide unambiguous structural evidence for the conversion of triglyceride soya oil to fatty acid methyl ester biodiesel, with the appearance of the methoxy carbon and disappearance of glycerol carbons serving as definitive markers of successful transesterification.

**Figure 6:**
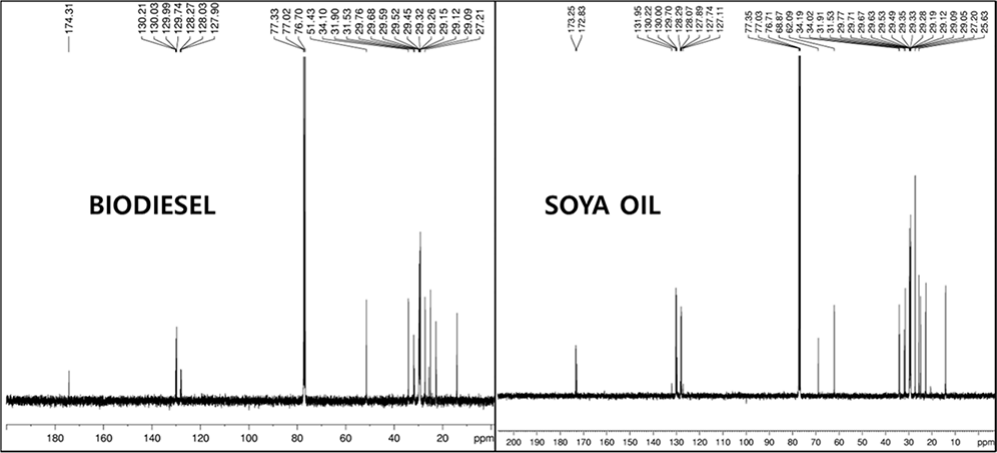
Comparative ^13^CNMR spectrum of soya oil and its derived biodiesel.

NMR analysis confirms that the transesterification of soybean oil to biodiesel was successfully completed, ensuring a predictable viscosity, oxygen content, and ignition behavior in the ternary fuel blend.

### 2.3 Experimental Protocol and Test Setup

Experimental testing was performed to assess the diesel engine performance and combustion and exhaust pollution characteristics, using a single-cylinder four-stroke engine, Kirloskar TV1, with a maximum power output of 5.2 kW at the engine speed of 1500 rpm. The engine possesses a constant compression ratio of 17.5, an injection pressure of 210 bar, and an injection timing established at 25° before the top dead center (bTDC). The test setup properties are outlined in Table 3.

**Table 3:** Main Specifications: Measurement Ranges of the Test Equipment

Parameter	Specification	
Engine brand and type	Kirloskar TV1	
Stroke	Four-stroke	
Total displacement	0.661 L	
Connecting rod length	234 mm	
Start of injection	0–25° bTDC	
Bore/stroke	87/110 mm	
Dynamometer cooling type	Water-cooled	
Dynamometer load indicator	Digital, 0–50 kg	
Dynamometer type	Eddy current	
Compression ratio	17.5:1	
Maximum power	5.2 kW at 1500 rev/min	
Crank angle resolution	Resolution 1°	
Maximum speed	5500 rpm	
Pressure sensor	0–210 bar ±0.5	
Pollutant emission	Specification	
Exhaust analyzer	HC	0–20000 ppm ± 1
	CO_2_	0–21.0% ± 0.1
	NO_*x*_	0–10000 ppm ± 1
	CO	0–10.0% ± 0.001
	O_2_	0–21.7% ± 0.01

The test apparatus for the experiments is shown in Figure 7. Pressure data were recorded by a transducer mounted within the cylinder. Combustion data including in-cylinder pressure (ICP) and net heat release rate (NHRR) relative to crankshaft position are taken from the engine via a data logger. All of the measurement apparatus was calibrated, and the engine was run until the engine temperature was stable before collecting the test data. Each test was performed thrice to make sure the obtained data was meaningful, and the average of these data was taken for visual displays of combustion, performance, and emission parameters.

**Figure 7:**
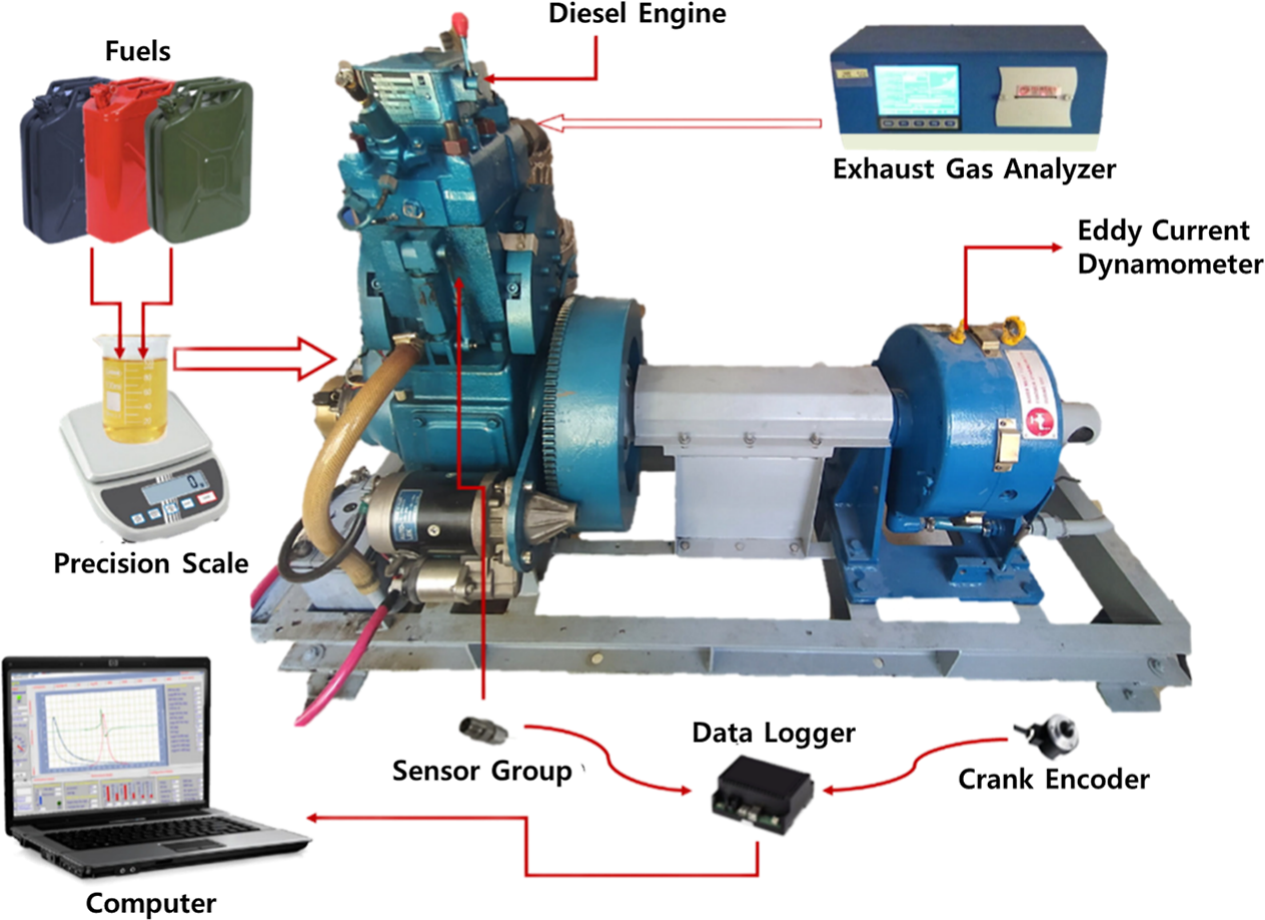
Visual demonstration of the experimental setup.

### 2.4 Error Analysis

Evaluations are made of device calibrations and operations within a specific range. Each of the variables under investigation has a correlation with the frequency of errors in the equipment under study. The total uncertainty can be calculated by adding the contributions from the instruments and errors in experiments. One crucial way in this study is the root-mean-square method, which calculates the overall level of uncertainty in the engine results.^41^ Error propagation was carried out using the root-sum-square method, where the total uncertainty of each calculated parameter was obtained by combining the uncertainties of their directly measured quantities. For a given performance parameter U expressed as a function of independent measured variables *x*_1_, *x*_2_, *x*_3_, ..., *x*_*n*_, the combined uncertainty was calculated using eq 1.

1 $$\Delta U=\sqrt{{\left(\frac{\partial U}{\partial x_{1}}\Delta x_{1}\right)}^{2}+{\left(\frac{\partial U}{\partial x_{2}}\Delta x_{2}\right)}^{2}+\cdot \cdot \cdot +{\left(\frac{\partial U}{\partial x_{n}}\Delta x_{n}\right)}^{2}}$$

The equation calculates the overall uncertainties for the given parameters, including brake power (BP), BSFC, BTE, ICP, NO_*x*_, CO_2_, CO, and HC under a particular operation condition. BP uncertainty was calculated from load and speed measurement errors. BSFC uncertainty was obtained by combining the uncertainties of BP and fuel consumption, while BTE uncertainty was calculated using the uncertainties of BP and BSFC. The percentage error of each parameter is calculated as follows: ±1%, ±1%, ±1%, ±0.1%, ±0.29%, ±0.95%, ±0.3%, and ±0.5%. Using the root-mean-square equation from eq 2, the overall uncertainty in percentage is ±2.09%.

2 $$\Delta U=\sqrt{BP^{2}+BSFC^{2}+BTE^{2}+ICP^{2}+N{O_{x}}^{2}+CO_{2}^{2}+CO^{2}+HC^{2}}=\pm 2.09\%$$

## 3 Result and Discussion

### 3.1 Combustion Parameters

The peak ICP is determined by the quantity of fuel participating in premixed combustion, which depends on the ignition delay duration and the atomization properties of the injected fuel.^42^ ICP and NHRR against crank angle degree (CAD) of D2, BD fuels, and BPro with MWCNT at concentrations of 40, 60, and 80 ppm per liter under different loads (Idle, 1.3, 3.1, and 4.6 bar BMEP) are illustrated in Figure 8. All fuel types show similar ICP curve shapes, as evident from the graphs. Findings from the graphs show that BD had the highest peak ICP in all engine loads when compared to engine operation with D2. Faster pressure decline of BD after the peak indicated a more intense combustion process, resulting in an 8.21 bar increase in ICP at 3.1 bar BMEP. BD exhibits a lower heating value and cetane number and higher viscosity and density compared to D2. The lower cetane number increased the ignition delay, allowing a larger amount of fuel to accumulate in the combustion chamber before the start of combustion. When ignition occurred, this accumulated mixture underwent rapid premixed combustion, which resulted in a sharper heat release and consequently higher peak ICP and NHRR. At full load, the peak ICP for BPro + CNT80 was 57.7 bar, which is approximately 6.8% lower than that of D2. For the other nanofuel blends, peak ICP values of 56.22 and 48.9 bar were recorded for BPro + CNT60 and BPro + CNT40, corresponding to reductions of 10% and 14.7%, respectively. MWCNT incorporation into the ternary blend resulted in significant peak pressure reduction and improved premixed combustion performance versus BD and D2. Peak ICP decreased for ternary fuels as a consequence of lower combustion temperatures and prolonged ignition delays, which retarded combustion timing bTDC into the expansion phase. The reason for this reduction can be the fact that MWCNTs in the fuel improved the ignition properties of the fuel, leading to better combustion performance, possibly thanks to their unique properties, including high surface area, thermal conductivity, and catalytic activity, which enhanced fuel evaporation, air–fuel mixing, and combustion completeness. In other words, combustion becomes more efficient, but without producing excessively high peak pressures. The presence of propanol in the fuel contributed to a lower pressure curve due to the thermal quenching effect associated with its high latent heat of evaporation. Moreover, the low cetane number of propanol along with biodiesel prolonged ignition delay and promoted greater fuel accumulation, ultimately reducing peak ICP. The average peak pressure difference values for BD, BPro + 40CNT, BPro + 60CNT, and BPro + 80CNT compared to petrodiesel were 5.50 bar, −8.21 bar, −5.38 bar, and −3.74 bar, respectively. The average percentage differences (mean percentage difference calculated across all tested engine loads) for the tested fuels BD, BPro + 40CNT, BPro + 60CNT, and BPro + 80CNT relative to petrodiesel were 10.27%, −14.74%, −10.07%, and −6.86%, respectively.

**Figure 8:**
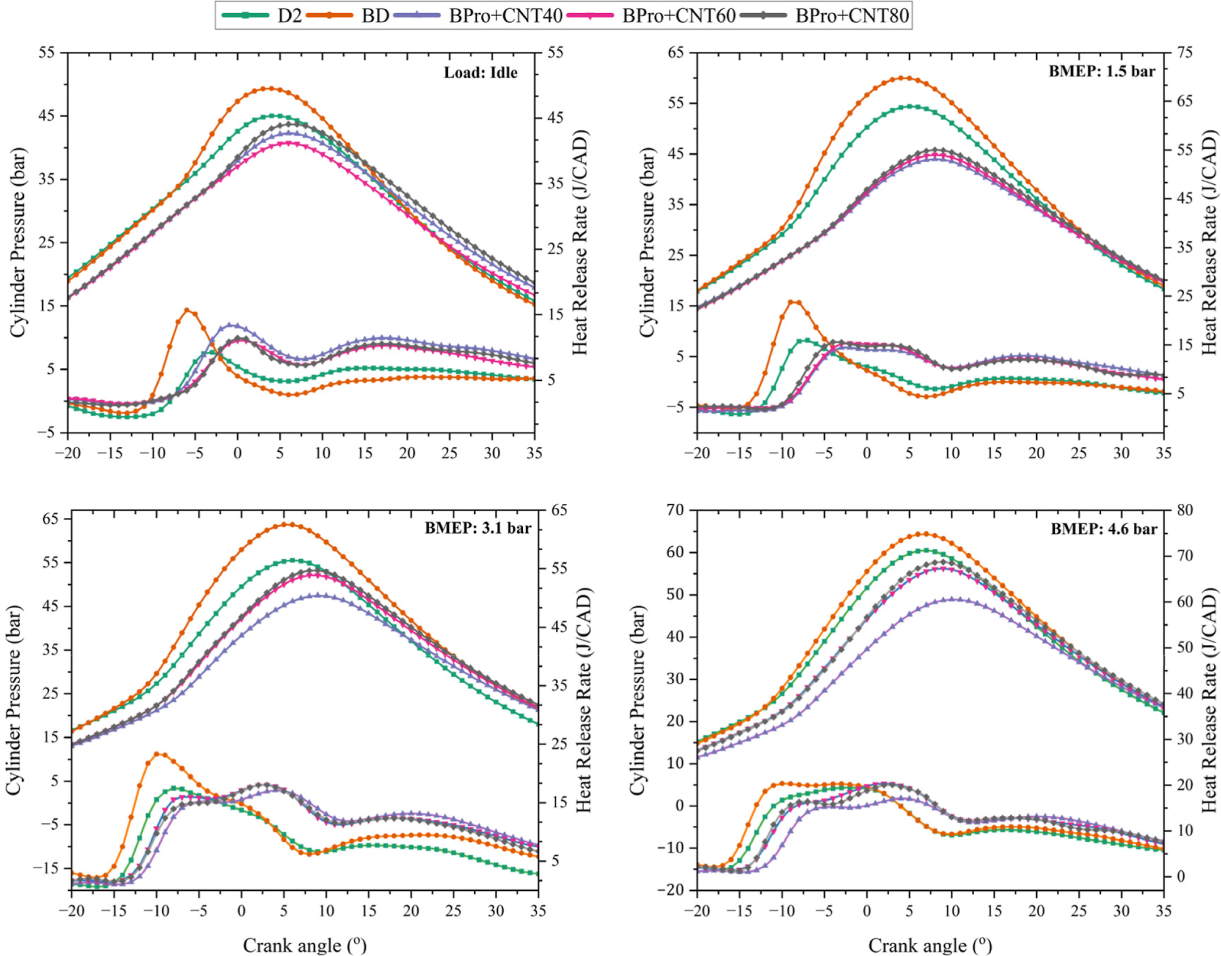
Variation of the peak NHRR and ICP versus crank angle of test blends with the loading of the MWCNT additive.

The highest NHRR during the experiments was produced by BD with 23.78 J/CAD, representing an increase of 7.82 J/CAD compared to that of D2. D2 showed a modest NNHRR peak, reflecting a controlled combustion process. Ternary blends with the addition of MWCNTs exhibited a higher peak NHRR compared to D2. Among the nanofuel blends, BPro + CNT40 exhibited the lowest peak NHRR, averaging 16.00 J/CAD, corresponding to a 7.62% enhancement relative to the reference fuel. The amount of NHRR was increased as the CNT content increased in the blend. Additionally, average NHRR values of 16.22 and 16.28 J/CAD were recorded for the BPro + CNT60 and BPro + CNT80 blends, respectively. Spray characteristics of the fuel sample increased with the MWCNT addition to the ternary blends, thanks to their very small particle size, which has quite high thermal conductivity and surface area. These catalytic properties increased the heat transfer between particles and the fuel droplet; thus, the evaporation rate increased. Considering the simultaneous increase in NHRR and the reduction in peak ICP, this can be explained by the combined influence of propanol and MWCNTs in the ternary blend. Propanol possesses both a high latent heat of vaporization and a lower cetane number, which leads to charge cooling during injection, prolonged ignition delay, and a shift of combustion phasing toward later crank angles, thereby reducing peak temperature and ICP. Conversely, MWCNTs exhibit exceptionally high thermal conductivity and catalytic surface activity, which enhance local heat transfer within fuel droplets, promote microscale oxidation reactions, and improve air–fuel mixing. These effects accelerate the premixed combustion phase and increase the NHRR despite the overall reduction in peak ICP. Consequently, the observed increase in NHRR accompanied by a decrease in peak ICP reflects a synergistic interaction in which propanol controls macroscopic thermal and phasing effects, while MWCNTs enhance microscopic reaction kinetics, leading to more efficient but less pressure-intensive combustion. Furthermore, the inherent oxygen content in both biodiesel and propanol enhanced combustion by acting as a catalyst. The start and duration of combustion ignition were postponed for the ternary blends. Nevertheless, MWCNT incorporation initially can result in an increase in ignition delay periods but then accelerates the combustion due to enhanced fuel characteristics and modified combustion behavior. The average NHRR difference values for BD, BPro + 40CNT, BPro + 60CNT, and BPro + 60CNT compared to petrodiesel were 5.24, 0.45, 0.67, and 0.73 J/CAD, respectively. The average percentage differences for the tested fuels BD, BPro + 40CNT, BPro + 60CNT, and BPro + 80CNT relative to petrodiesel were 39.02%, 7.62%, 5.87%, and 6.73%, respectively.

### 3.2 Performance Parameters

The variation in BTE with varying loading conditions for all test fuels is shown in Figure 9. Efficiency indicates the degree to which fuel energy transforms into productive work.^43^ BD produced the least BTE across all of the load operations due to its higher kinematic viscosity and density and lower heating value than the remaining fuels. This resulted in inadequate atomization during the vaporization process within combustion. MWCNT addition to BPro fuel increased BTE for all of the load operations. The highest BTE value of 38.5% was obtained with the BPro + CNT80 blend, followed by 37.3% for BPro + CNT60, 36.8% for BPro + CNT40, 35.6% for D2, and 34.1% for BD. MWCNTs in BPro fuel enhanced the thermal conductivity of the fuel mixture. The improvement in BTE can be attributed to the high thermal conductivity and large surface area of MWCNTs, which enhanced heat transfer and promoted more complete oxidation during the main combustion phase. The presence of MWCNTs also improved the microscale air–fuel blend and provided active surface sites for combustion reactions, leading to faster and more efficient energy release. Additionally, the oxygen-rich nature of propanol and biodiesel in ternary blends, coupled with the lower boiling point of propanol, resulted in reduced heat losses during combustion.^44^ Moreover, the elevated surface area to volume ratio of the MWCNTs facilitated droplet size reduction during ternary fuel injection processes. These mechanisms collectively resulted in improved combustion characteristics and enhanced efficiency. The average BTE percentage differences for the tested fuels BD, BPro + 40CNT, BPro + 60CNT, and BPro + 80CNT relative to petrodiesel were −7.11%, 9.09%, 11.27%, and 11.79%, respectively. Therefore, ternary blends incorporating propanol with MWCNTs demonstrated superior efficiency in generating usable power compared with BD and diesel.

**Figure 9:**
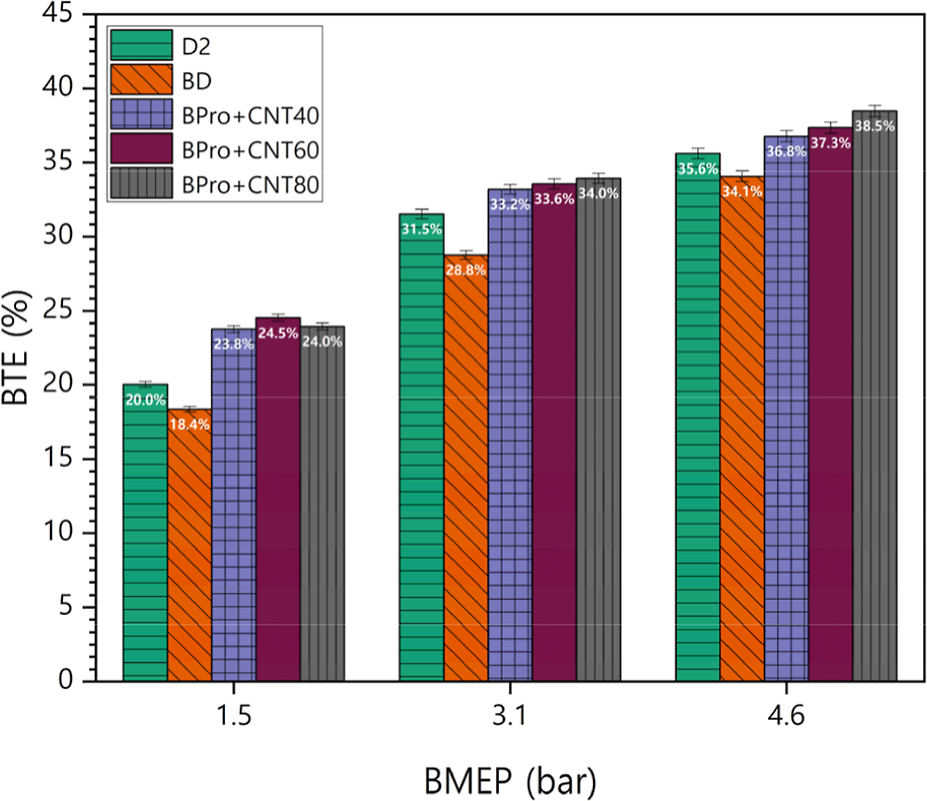
BTE variation of test blends with the loading of the CNT additive.

The variation in the BSFC under different loading conditions for all test fuels is shown in Figure 10. The trend obtained with BSFC results is the opposite of that commented for BTE, which is consistent. BD exhibited the highest BSFC across all loads due to its elevated viscosity and calorific value. MWCNT incorporation into the blend achieved substantial BSFC reduction proportional to its concentration in the fuel mixture due to its energy content. The BPro + CNT80 attained the lowest BSFC of 0.282 kg/kWh, which was lower than both biodiesel and diesel values. MWCNTs acted as effective combustion catalysts and offered remarkable thermal conductivity performance. The enhancement reflects MWCNTs’ ability to optimize combustion through catalytic activity and enhance fuel injection through improved atomization due to superior surface area properties. Rising MWCNT concentrations in fuel mixtures delivered improved combustion efficiency by creating finer fuel droplet sprays through improved atomization, which enabled better air–fuel mixing and more thorough oxidation processes. Also, the MWCNTs provided better fuel characteristics with improved ignition characteristics. MWCNTs enhanced flame propagation consistency, leading to more stable combustion and a reduced risk of incomplete burning or combustion irregularities that cause energy losses. As the MWCNT concentration increased, more controlled and stable combustion allowed the engine to convert fuel energy into useful work more efficiently. Through acceleration of complete combustion processes, nanoparticles optimized fuel energy utilization for useful work, directly enhancing BTE while minimizing BSFC. The average BSFC difference values for BD, BPro + 40CNT, BPro + 60CNT, and BPro + 80CNT compared to those of petrodiesel were 65.84 g/kWh, −14.62 g/kWh, −20.21 g/kWh, and −20.63 g/kWh, respectively. The average percentage differences for the tested fuels BD, BPro + 40CNT, BPro + 60CNT, and BPro + 80CNT relative to petrodiesel were 22.62%, −3.58%, −5.37%, and −6.04%, respectively.

**Figure 10:**
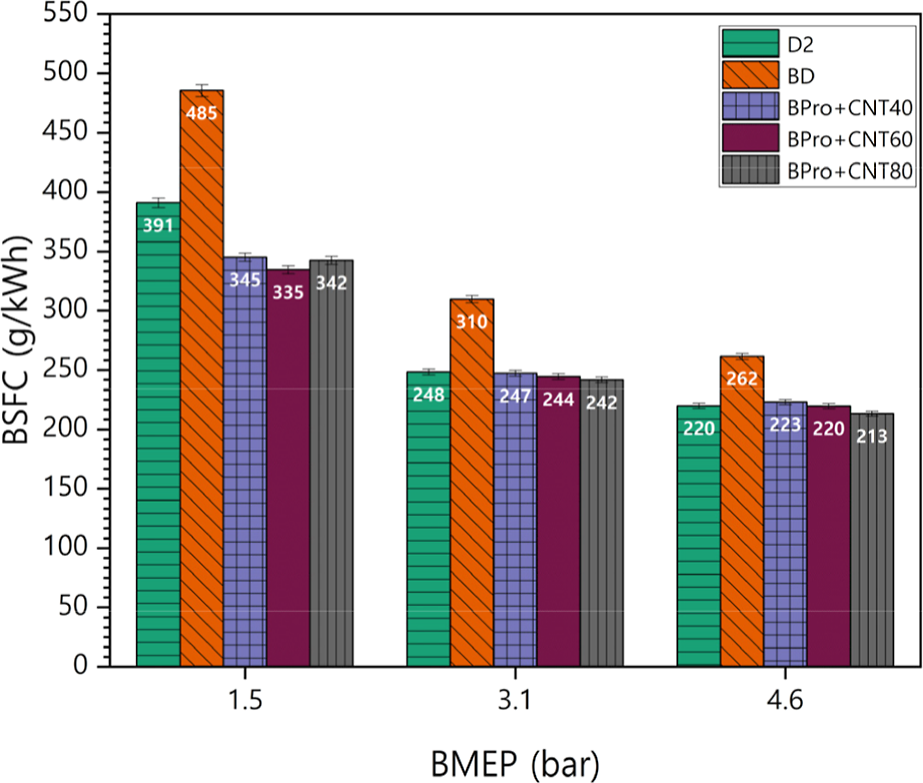
BSFC changes of test blends under varying loads with the CNT additive.

### 3.3 Exhaust Gaseous Emissions

Figure 11 displays the variations in HC emissions of the test fuels under varying loads with the MWCNT additive in the engine exhaust. According to the figure, HC emissions escalated under higher engine loads, as increased fuel–air ratios promote combustion inefficiencies. BD blends demonstrated reduced HC emissions attributed to the oxygen content within their molecular composition. In contrast, ternary fuel mixtures exhibited elevated HC emissions, relative to conventional diesel, across all operational parameters. The increase in HC emissions observed for the MWCNT-enhanced BPro blends can be attributed to the combined influence of the propanol and nanoparticle behavior. Propanol has a high latent heat of vaporization and lower volatility compared to diesel, which reduces local mixture temperatures during vaporization^45^ and promotes wall quenching, particularly in colder boundary-layer regions. Additionally, the lower cetane number of propanol extends the ignition delay, allowing a greater fraction of unburned or partially oxidized fuel to persist near the combustion chamber walls. Although MWCNTs generally enhance combustion through improved thermal conductivity and flame propagation, slight nanoparticle agglomeration can lead to localized heat sinks that further lower the temperature in certain zones. As a result, the catalytic effect of MWCNTs is not sufficient to fully counteract the cooling and phasing effects introduced by propanol, leading to elevated HC emissions in the exhaust. Jayapal and Radhakrishnan^46^ also reported higher HC emissions with inclusion of propanol to biodiesel and diesel. Similarly, MWCNTs showed increased HC emission in a research by Sathish et al.^47^ The average HC difference values for BD, BPro + 40CNT, BPro + 60CNT, and BPro + 80CNT compared to petrodiesel were −16.25 ppm, 2.25 ppm, 8.00 ppm, and 3.75 ppm, respectively. The average percentage differences for the tested fuels BD, BPro + 40CNT, BPro + 60CNT, and BPro + 80CNT relative to petrodiesel were −53.34%, 8.24%, 26.66%, and 13.95%, respectively.

**Figure 11:**
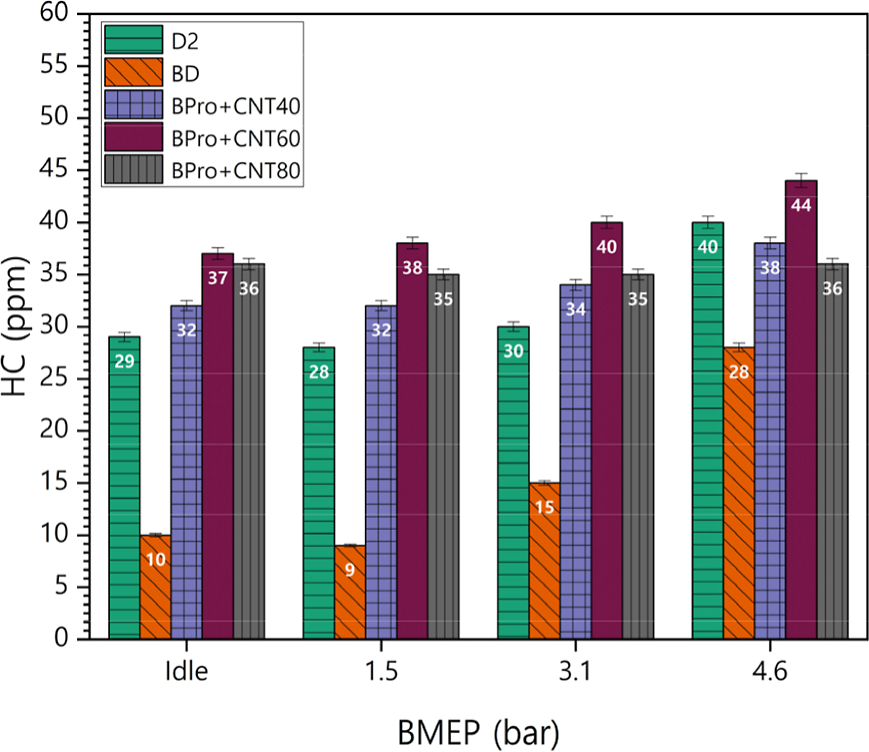
Variations in HC emissions of test blends under varying loads with the CNT additive.

Variations in CO_2_ emissions of the test fuels under varying loads with the MWCNT additive in the engine exhaust are shown in Figure 12. CO_2_ emissions increased proportionally with engine load, indicating complete HC fuels facilitated by improved air–fuel mixing. D2 and BD produced the highest CO_2_ emissions compared to those of ternary blends. When the amount of MWCNTs was raised from 40 to 80 ppm, the amount of CO_2_ released from the engine was further decreased. The reduction in CO_2_ emissions is attributed to the combined effects of lower BSFC due to improved BSFC and the lower carbon content of the oxygenated components of both biodiesel and propanol, resulting in reduced carbon released per unit work output. At higher engine loads, the overall combustion temperature and reaction rate increase, promoting more complete oxidation in the main combustion zone, which results in higher CO_2_ emissions. However, higher loads also lead to locally fuel-rich and near-wall regions, where oxygen availability is limited and quenching occurs, causing an increase in HC emissions. Therefore, the simultaneous rise in CO_2_ and HC does not indicate contradictory combustion behavior but rather reflects the coexistence of efficient bulk combustion and localized incomplete oxidation. Furthermore, biodiesel and propanol offer environmental benefits, as their biological production processes create a carbon-neutral cycle that does not contribute to net greenhouse gas accumulation. The average CO_2_ percentage differences for BD, BPro + 40CNT, BPro + 60CNT, and BPro + 80CNT compared to petrodiesel were −3.16%, −13.28%, −16.45%, and −18.72%, respectively.

**Figure 12:**
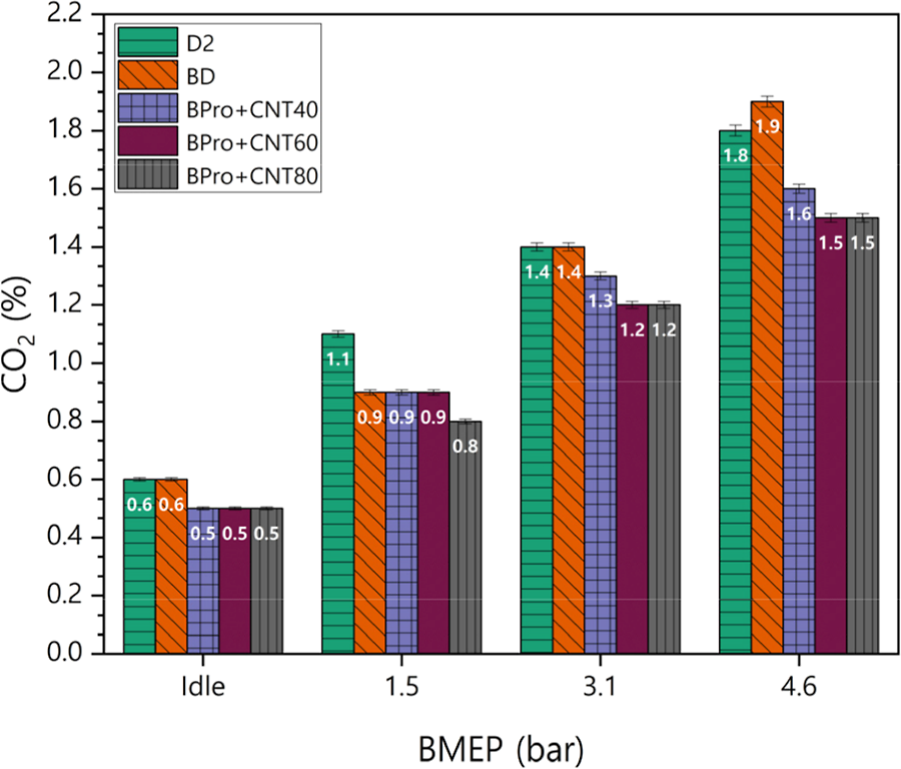
Variations in CO_2_ emissions of the test fuels under varying loads with the MWCNT additive.

CO formation results from incomplete combustion caused by insufficient air entrainment, inadequate combustion processes, and poor air–fuel mixing.^48^ Variations in the CO emissions of the test fuels under varying loads with the CNT additive in the engine exhaust are shown in Figure 13. Lower postcombustion temperatures elevate CO emissions by disrupting oxidation reactions.^43^ From the findings, the CO emissions of diesel and biodiesel were relatively higher than the emissions recorded from ternary mixtures with MWCNTs at some loads. MWCNT addition also contributed to higher CO emissions due to the carbon content present in the nanotubes. It should be noted that the CO emission results exhibited load-dependent variation, as the formation of CO is highly sensitive to local combustion temperature and oxidation timing. The competing effects of propanol’s evaporation cooling and the catalytic activity of MWCNTs can shift the balance between complete and partial oxidation differently at each load point. Therefore, the CO behavior reflects the combined influence of heat release phasing and near-wall quenching effects.

**Figure 13:**
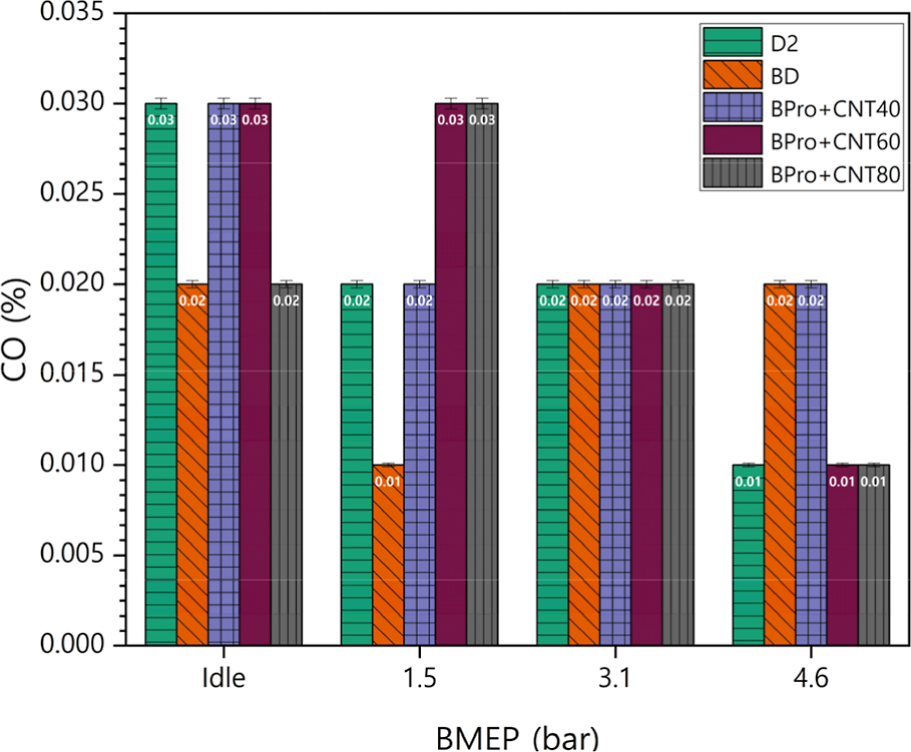
Variations in CO emissions of the test fuels under varying loads with the MWCNT additive.

Figure 14 shows the variations in NO_*x*_ emissions of the test fuels under varying loads with the MWCNT additive in the engine exhaust. An increase in NO_*x*_ emissions with rising BMEP was observed for all fuels, attributed to higher fuel–air ratios, which promote larger fuel volumes undergoing combustion, elevating gas temperatures and thereby enhancing NO_*x*_ formation. Among test samples, D2 fuel exhibited the highest NO_*x*_ emissions, which correlates with its higher density property, which enhances combustion efficiency and increases gas temperatures within the combustion chamber. BD demonstrated the second-highest NO_*x*_ levels during experiments due to its elevated viscosity and density, producing maximum peak pressure during combustion and resulting in increased in-cylinder temperatures.^49^ NO_*x*_ emissions decreased significantly for blends containing MWCNTs, with reduction levels being approximately equivalent. Propanol addition to blends increased the oxygen content while reducing the cetane index of fuel mixtures. The reduction in NO_*x*_ emissions can be attributed mainly to the influence of a high latent heat of vaporization of propanol, resulting in lower in-cylinder temperature and longer ignition delay. These combined effects suppressed thermal NO_*x*_ formation. The role of MWCNTs was secondary because they have high thermal conductivity and surface activity that helped stabilize combustion and maintain efficiency under these cooler combustion conditions. Thus, propanol primarily contributed to the NO_*x*_ reduction, while MWCNTs mitigated potential efficiency loss due to lower flame temperature. Furthermore, more efficient energy conversion indicated by BSFC improvements for nanofuels required additional oxygen molecules interacting with fuels, leaving fewer oxygen molecules available in the environment. The average NO_*x*_ difference values for BD, BPro + 40CNT, BPro + 60CNT, and BPro + 80CNT compared to petrodiesel were −10.00 ppm, −53.25 ppm, −56.00 ppm, and −52.00 ppm, respectively. The average percentage differences for the tested fuels BD, BPro + 40CNT, BPro + 60CNT, and BPro + 60CNT relative to petrodiesel were −8.77%, −40.67%, −42.39%, and −38.29%, respectively.

**Figure 14:**
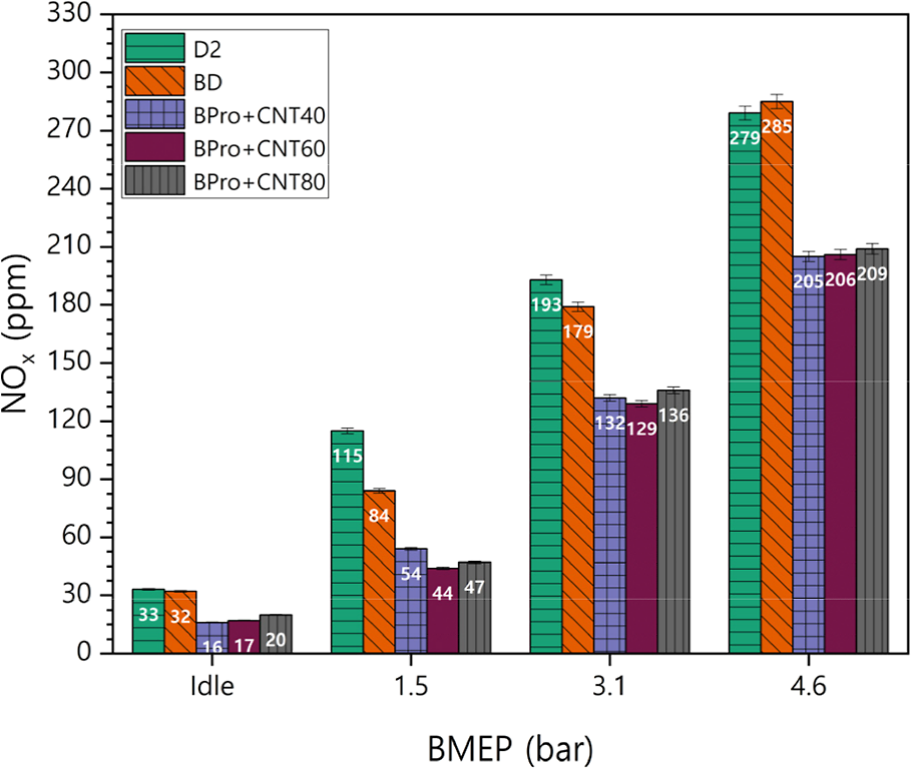
Variations in NO_*x*_ emissions of the test fuels under varying loads with the MWCNT additive.

## 4 Conclusions

In this study, the effects of ternary blends consisting of biodiesel (10% v/v), propanol (10% v/v), and diesel fuel (80% v/v) with MWCNT concentrations of 40 ppm, 60 pmm, and 80 ppm were examined on a monocylinder diesel engine. The diesel-fueled internal combustion engine performance was analyzed and compared with that of conventional diesel and pure biodiesel. Following a comprehensive evaluation of combustion, performance, and emission characteristics, the subsequent conclusions were obtained.

1. The incorporation of nanoparticles enhanced the blend’s properties, resulting in a more sustained energy release, improved thermal conductivity, and higher efficiency.
2. Compared to petrodiesel, HC emissions show an increase for BPro + 40CNT, BPro + 60CNT, and BPro + 80CNT fuels by an average of 8.24%, 26.66%, and 13.95%, respectively. The rise in HC emissions can be attributed to the high enthalpy of vaporization of propanol, which favors a lower combustion temperature and consequently promotes incomplete combustion. Likewise, CO emissions showed no clear trend but increased on average. Contrarily, CO_2_ emissions decreased by an average of 13.28%, 16.45%, and 18.72% for BPro + 40CNT, BPro + 60CNT, and BPro + 80CNT fuel blends, respectively, due to lower flame temperatures and partial oxidation, which decreased complete carbon conversion.
3. NO_*x*_ emissions were reduced by an average of 40.67%, 42.39% and 38.29% for BPro + 40CNT, BPro + 60CNT, and BPro + 80CNT fuel blends, respectively, because of the increased thermal conductivity brought about by CNT addition and, mainly, the high enthalpy of vaporization of propanol, which decreases the peak combustion temperatures and thus limits temperature-dependent NO_*x*_ formation.
4. BSFCs were the lowest, similarly for ternary blends with MWCNTs, with improvement by up to an average of 6.04%, when compared to petrodiesel.
5. The peak ICP was lessened while peak NHRR increased, showing more sustained energy release and constant combustion, which facilitates more uniform energy transfer to the piston over the power stroke. As a result, this contributes to an improvement in the engine’s BTE.
6. BTE values were the highest for MWCNT-enhanced blends with the highest of 38.5% for the ternary blend with 80 ppm MWCNTs. BTE is improved by an average of 11.79%.

Overall, the findings demonstrate that the ternary diesel–biodiesel–propanol blend enriched with MWCNTs can improve combustion stability, enhance brake thermal efficiency, and significantly reduce NO_*x*_ compared with conventional diesel. Among the tested fuels, the BPro + CNT80 nanofuel exhibited the most favorable performance, achieving the highest BTE and the lowest NO_*x*_ and CO_2_ emissions while maintaining acceptable combustion characteristics. However, previous studies have indicated that the use of nanoparticles in fuel systems may raise environmental and health considerations due to their ultrafine size and high surface reactivity, which could potentially facilitate inhalation and accumulation in the respiratory system if released into the exhaust stream.^50^ Although nanoparticle emissions were not directly measured in this study, the responsible implementation of nanofuels in practical applications may require the use of appropriate exhaust after-treatment or nanoparticle capture systems, such as diesel particulate filters or electrostatic precipitators, to mitigate any potential nanoparticle release. For future research, it is recommended to investigate long-term engine durability with repeated nanofuel usage, optimize nanoparticle concentration to balance performance and cost, and integrate nanoparticle emission control systems (e.g., electrostatic precipitators) to mitigate potential nanoparticle release in exhaust streams.

## Abbreviations

^13^C NMR: Carbon-13 nuclear magnetic resonance
^1^H NMR: Proton nuclear magnetic resonance
BD: Pure biodiesel
BP: Brake power
BSFC: Brake-specific fuel consumption
bTDC: Before top dead center
BTE: Brake thermal efficiency
BPro: Diesel–biodiesel–propanol blend (80:10:10 by volume)
BPro + CNT40: BPro blend with 40 ppm MWCNTs
BPro + CNT60: BPro blend with 60 ppm MWCNTs
BPro + CNT80: BPro blend with 80 ppm MWCNTs
CIEs: Compression ignition engines
CAD: Crank angle degree
CO: Carbon monoxide
CVD: Chemical vapor deposition
D2: Ultra low sulfur diesel
DTA: Differential thermal analysis
EVs: Electrical vehicles
FAME: Fatty acid methyl esters
FTIR: Fourier transform infrared
HC: Hydrocarbons
HCCI: Homogeneous charge compression ignition
ICP: In-cylinder pressure
MWCNTs: Multi-walled carbon nanotubes
NHRR: Net heat release rate
NO*x*: Nitrogen oxides
PM: Particulate matter
RCCI: Reactivity-controlled compression ignition
SEM: Scanning electron microscopy
TEM: Transmission electron microscopy
TG: Thermogravimetric
